# A New Insight into Biospeckle Activity in Apple Tissues

**DOI:** 10.3390/s19030497

**Published:** 2019-01-25

**Authors:** Christelle Abou Nader, Jean-Michel Tualle, Eric Tinet, Dominique Ettori

**Affiliations:** Laboratoire de Physique des Lasers, CNRS UMR 7538, Institut Galilée, Université Paris 13, 99 Av. J. B. Clément, F-93430 Villetaneuse, France; tualle@univ-paris13.fr (J.-M.T.); eric.tinet@univ-paris13.fr (E.T.); ettori@univ-paris13.fr (D.E.)

**Keywords:** biological sensing, food monitoring, speckle, light scattering, biospeckle activity, diffusion

## Abstract

The monitoring and characterization of agricultural products before harvest or during ripening, storage, and shelf life has recently been increasingly explored in the literature. The analysis of biospeckle activity has potential for the determination of the optimal harvest window, the monitoring of the fruit ripening process, and the detection of diseases and bruising. In this technique, the specimen is illuminated with coherent light and speckle intensity fluctuations are analyzed using diverse methodologies. Prior work shows that biospeckle activity is strongly correlated to physiological indexes conventionally used to evaluate fruit texture and composition. Here, we scrupulously investigate the biospeckle activity of Gala apple fruits during postharvest stages. We simulate realistic conditions for shelf-life monitoring, namely an unknown history of the fruit and storage in an uncontrolled atmosphere. Scattering spot images are acquired with multiple exposure times using a simple optical setup. The contrast, reflecting biospeckle activity, is computed after eliminating inhomogeneous zones. The results show, for the first time, speckle activity at short time scales. The retrieved correlations between speckle parameters and the ratio of apples’ firmness to their soluble solids content reveal significant links despite the unknown fruit’s origin, harvest date, and storage history.

## 1. Introduction

Extensive research has reported on the monitoring and study of agricultural products due to the increasing consciousness of quality in the food sector. As a matter of fact, whether the product is heading straight to the consumer, being exported, or being used as an intermediate in a food production chain, it is of major interest to monitor its quality evolution during maturation, storage, and shelf life [1]. Accordingly, inspections based on quality features are performed in order to achieve grading and sorting, particularly of harvested fruits before packaging or export. These quality attributes include external appearance, such as size, shape and color. Texture is also one of the main aspects defining fruit quality. Textural properties can be estimated through sensory analysis, performed by a trained panel, or through instrumental measurements using fundamental or empirical methods. Fundamental tests include the measurement of different well-known parameters such as Poisson’s ratio, Young’s modulus, and the shear modulus [2]. However, empirical methods like puncture, compression, and extrusion are more commonly used for their simplicity, as they provide parameters that were found to be correlated with texture. Other key features for the evaluation of fruit quality include soluble solids content, firmness, starch content, titratable acidity, ethylene emission, and respiration rate [3,4]. Nevertheless, the evaluation of most of these quality attributes and physiological parameters remains time-consuming and costly. Hence, various promising optical methods have been tested for the inspection of fruit quality.

Given that changes in fruits’ attributes induce variations in the optical properties of their skin and inner flesh, multiple techniques sensitive to these changes have been considered. These methods, such as time-resolved reflectance spectroscopy [5], hyperspectral imaging [6,7,8], NIR spectroscopy [9,10,11], laser-induced light backscattering imaging [12], and chlorophyll fluorescence [13], could pave the way for fast, non-invasive, and low-cost monitoring, with a potential use directly in orchards. Most of these methods are used during pre-harvest stages, where optical properties tend to vary significantly, in order to determine the optimal harvest date.

In addition to changes in optical properties induced by attribute modifications, variations in fruits’ inner activity have also been reported [14]. This activity, often associated with the physical movement of cell walls and particles inside cells, was extensively studied via the analysis of intensity fluctuations of the speckle pattern, known as biospeckle [15]. In fact, biospeckle arises from the random interference of coherent light interacting with a scattering object. In the case of biological media, the intrinsic scattering particles, called scattering centers, often undergo some kind of movement. Light scattering by these moving particles causes phase shifts in the scattered light and changes the random interference pattern in time, inducing speckle intensity fluctuations or biospeckle activity. Changes in biospeckle activity were observed throughout the maturation process as well as disease manifestations [16,17]. A link between speckle intensity fluctuations and fruit quality was confirmed in multiple studies [18,19]. Typically, the biospeckle activity is assessed through analysis of the speckle contrast at one exposure duration, the moment of inertia, or the correlation between an image taken as a reference speckle pattern, and a sequence of the following frames acquired over a time series [14]. However, to our knowledge, no previous studies have yet investigated the relationship between fruit attributes and the evolution of biospeckle activity over short time scales ranging from 1.5 to 98 ms. Furthermore, very few works have examined biospeckle activity during shelf life [17,20,21] or under realistic conditions where the history, harvest date, and origin of the fruit are unknown.

In the present study, we monitor the evolution of the biospeckle activity of a batch of apple fruits over short time scales, through contrast measurements. The Gala apples were bought from a local market on different days. A NIR laser source is used to illuminate an apple slice, and scattering spot images are acquired over multiple exposure durations in trans-illumination geometry using a CCD camera with an objective. Contrast values are computed from a ring zone after localizing the center of the scattering spot and removing inhomogeneous regions. Parameters describing the evolution of the contrast over different exposure times, as well as fruits’ physiological attributes, namely firmness and soluble solids content (SSC), are assessed for each apple. The results reveal, for the first time, a correlation between parameters describing speckle fluctuations over very short time scales and fruit firmness. This correlation is improved when considering the firmness and SSC level concurrently via their ratio. These results highlight the contribution of the SSC level to speckle activity, along with that of the firmness. The present work reveals fast dynamics with very short characteristic times that could be related to rapid metabolic processes happening within the fruit.

## 2. Materials and Methods

### 2.1. Samples

A batch of 55 Gala (Malus x Domestica) apples was purchased from a local market, on different days over a two-month period, and kept at room temperature (20 °C) in an uncontrolled atmosphere in terms of air quality for various periods of time. These conditions are equivalent to those under which apples are preserved during their shelf life in a store, without prior knowledge of either their harvest date or storage history. All selected apples had no visible damage or blemishes. Each fruit was cut into a 12 mm thick slice that was immediately used for speckle testing. Firmness and SSC measurements were simultaneously performed on the remaining part of each apple. The experiments were carried out after different numbers of days following their purchase date. This was done to make sure that we have covered a sufficient range of textural properties, as fruits evolve from their best textural conditions (on their purchase date) to the final stages of their shelf life, when their quality usually tends to degrade. Note that all apples were not purchased on the same date in order to make sure that they do not originate from the same orchard.

### 2.2. Speckle Setup

**Figure 1:**
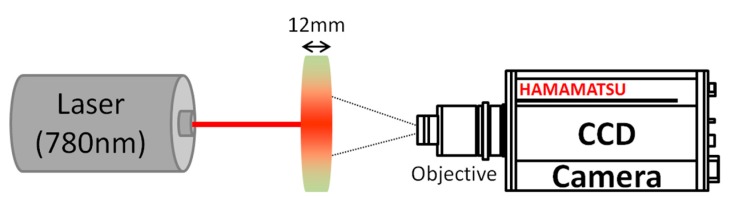
Schematic view of the speckle experimental setup. A 780 nm laser beam illuminates an apple slice. Scattering spot images are acquired using a CCD equipped with an objective.

The speckle experimental setup is presented in Figure 1. Light from a laser diode source (wavelength λ = 780 nm) illuminates a freshly cut apple slice (thickness =12 mm). The wavelength was specifically chosen in order to minimize the absorption of apple tissues [22]. Note that in this range of wavelength, apples’ absorption and reduced scattering coefficients do not show any significant change with respect to storage time [23] and that the penetration depth of such wavelength in fruit tissues is higher than the considered slice thickness [22]. Light scattered through the sample slab is collected by a CCD camera (Hamamatsu Photonics, Hamamatsu, Japan, C8484-05G01, 1344 × 1024 pixels, 6.45 µm × 6.45 µm pixel size), placed ~35 cm from the slab (~0.14 magnification, 216 pixels corresponding to 1 cm on the sample), with an objective set at an *f*-number of 11 in order to observe subjective speckle. The image of the observed side of the apple slice is slightly defocused in order to remove the contribution of sharp surface structures to the speckle pattern analysis. This defocusing was characterized by imaging the laser spot through tracing paper placed at the location of the observed apple side, leading to a 3.7 mm apparent diameter of the laser spot.

### 2.3. Speckle Measurements

Images of the scattering spot for each apple slice are taken under different exposure durations *T*_exp_, ranging from 1.5 to 98 ms. To make sure that all images over the whole range of exposure durations have the same mean intensity, the laser’s intensity is automatically adjusted repeatedly, with a maximal power of 20 mW at shorter times. For each exposure duration, 40 images are averaged out: all images, from all exposure durations, are added up to form a global image of the diffuse light averaged intensity. On this image, an internally developed algorithm that detects circular patterns is used to identify the center of the scattering spot and eliminate large-scale inhomogeneous zones. This algorithm is based on the cylindrical symmetry expected from the averaged intensity: for each distance r (in pixels) from the scattering spot center, we extrapolate N(r)=4πr intensity values from the recorded data along the circle of radius r. We then extract the subset of γN(r) points (γ=0.25 in this study) with minimal dispersion (i.e., with a minimal difference between its maximal and minimal values). The set of all these extracted points will be considered for the contrast analysis of the speckle patterns, thus removing inhomogeneous zones. These zones could be the result of light scattering by certain inhomogeneous regions like the stem or seeds from the center of the slice and are suppressed from all acquired images. As a matter of fact, we chose to use subjective speckle in order to be able to have a spatial resolution granting the possibility to eliminate such inhomogeneous regions. This step is essential, as it allows us to collect information originating solely from apple flesh.

To evaluate speckle fluctuations, and thus the biospeckle activity that arises from moving scattering centers inside the sample, we chose the contrast as a parameter of interest. In fact, the contrast *C* is a function of the electric field temporal autocorrelation function *G*_1_ as [24,25]:

(1) $$C(T_{\exp})=\frac{1}{T_{\exp}}\sqrt{2\beta \int_{0}^{T_{\exp}}(T_{\exp}-\tau )\frac{G_{1}^{2}(\tau )}{G_{1}^{2}(0)}d\tau },$$

where *β* accounts for a reduction in contrast related to light depolarization and experimental conditions (objective *f*-number, pixel size, …). This justifies the contrast’s direct relationship with speckle fluctuations. Note that *G*_1_ is related to the scattering particle’s mean square displacement (MSD), which represents the deviation of a particle’s position with respect to a reference location over time.

For all scattering spot images acquired over the whole range of exposure durations, we compute the contrast over a ring zone with a 9.2 mm inner and a 9.4 mm outer diameter. This zone was chosen as a compromise for the signal to noise ratio that is limited by both the distance to the scattering spot center and the surface of the ring. Thereby, the contrast is calculated as follows [26]:

(2) $$C(T_{\exp})=\frac{\sigma (T_{\exp})}{\langle I(T_{\exp})\rangle },$$

where σ(*T*_exp_) is the standard deviation of the recorded speckle pattern, and 〈*I*(*T*_exp_)〉 is its mean intensity. *C*(*T*_exp_) allows an estimation of biospeckle activity and presents a simple means to assess inner activity using speckle temporal fluctuations. This parameter can take values between 0 and 1 in the case of a fully developed speckle under polarized illumination conditions [24]. A *C* value of 1 indicates that the speckle pattern is not blurred and therefore, an absence of activity. In this case, the scattering centers are static. A *C* value of 0 indicates a fully blurred speckle pattern meaning that the scattering centers are moving fast enough to blur all of the speckles. In practice, *C* values cannot exceed a $\sqrt{\beta }$ factor, and therefore would take values between 0 and $\sqrt{\beta }$. Thus, a contrast value of $\sqrt{\beta }$ would mean an absence of intrinsic activity in the sample. Our experimental conditions yield a value of $\sqrt{\beta }=0.54$ assessed using a calibrated medium having no intrinsic activity.

In summary, we compute, for each apple, the contrast as a function of the image exposure duration. In order to evaluate and compare the behavior of the contrast over shorter and longer exposure durations, two linear fits of the form *C*(*T*_exp_) = *aT*_exp_ + *b* are applied to contrast values corresponding to shorter times (*T*_exp_ between 1.5 and 20 ms) and longer ones (*T*_exp_ between 78 and 98 ms). The slope and intercept of both fits are (*a*_short_, *b*_short_), and (*a*_long_, *b*_long_), respectively.

### 2.4. Conventional Measurements

As the fruit’s textural properties and sweetness are key factors determining its appreciation by the consumer, the measurement of firmness and soluble solids content (SSC) is commonly used to evaluate fruits quality. Firmness is often assessed for the estimation of fruit softening. In this work, apple firmness, expressed in $\mathrm{kg}\cdot {\mathrm{cm}}^{-2}$, was determined using an electronic penetrometer (PCE Instruments, Southampton, UK). Each studied apple was peeled and penetration measurements were performed at three different equatorial locations on the apple using an 11.3 mm diameter point. The three firmness values are averaged out and their standard deviation is computed.

The SSC was measured using a portable refractometer (PCE-032, PCE Instruments, Southampton, UK). A drop of the filtrate of the apple pulp was placed onto the instrument’s prism, which measures the juice’s refractive index. SSC values are given using the Brix scale, which indicates the percentage (%) of dissolved solids in the solution. Note that typically, in fruit juice, sugars are the major soluble solids. Other materials, representing mainly a minor percentage of the dissolved solids, include organic and amino acids, soluble pectins, phenols, and minerals.

## 3. Results and Discussion

**Figure 2:**
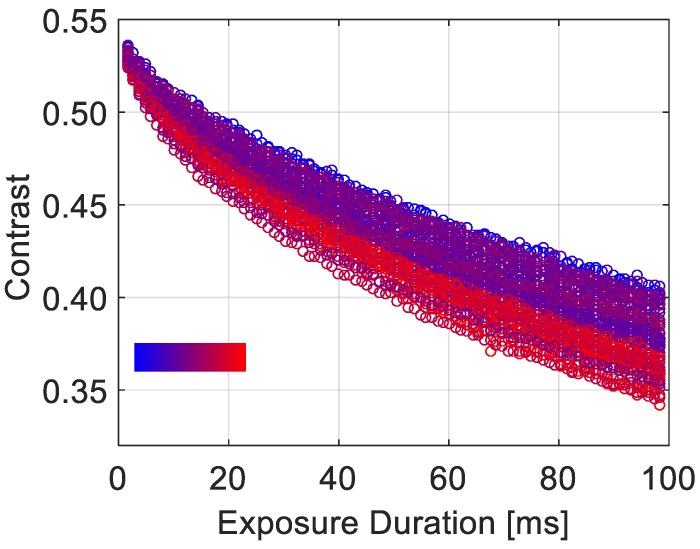
The contrast *C*, computed over rings with a 9.2 inner and 9.4 mm outer diameter, as a function of the exposure duration *T*_exp_ for all 55 samples. Different colors correspond to different apples. The chosen color range is scaled as a function of the ratio of the firmness to the soluble solids content (SSC) level: blue and red correspond to lower and higher levels, respectively.

Previous works have essentially evaluated apples’ biospeckle activity by calculating the correlation between the first frame of a time series and a frame taken after about 4 or 14 s [14,27]. In this study, we chose to look closer into faster dynamical behavior with shorter characteristic times, and test the correlation to apples’ physiological features. Since we had no precise knowledge of scattering particles’ dynamics in apple tissues over short times, we chose to explore a rather large range of exposure durations. The contrast over the whole chosen range (1.5 to 98 ms) for all studied apples is represented in Figure 2.

Contrast profiles show similar trends for all samples. As speckle images are acquired with longer exposure durations, a decrease in contrast values is observed. This behavior is typically representative of a medium where scattering particles are undergoing some kind of motion, with dynamic characteristic times in the range of the considered exposure duration. This proves that light is indeed scattered by intrinsic particles diffusing rapidly, with short characteristic times.

When comparing different apples, one could examine the contrast at a precise exposure duration, adapted to differentiate the observed dynamics. Here, we choose the contrast *C*_98ms_ of images acquired with a 98 ms exposure, where we have the largest range of contrast variation among apples. We also consider the slopes and intercepts *a*_short_, *b*_short_, *a*_long_, and *b*_long_ of the linear fits corresponding to shorter and longer exposures, evaluated for all apples. These parameters could differentiate dynamics based on different characteristic times. Concerning conventional firmness and SSC measurements, undertaken on the same apples that were optically tested, values ranged from 2.9 to 7.7 kg·cm^−2^ for firmness and from 9.8% to 15.8% for SSC.

**Figure 3:**
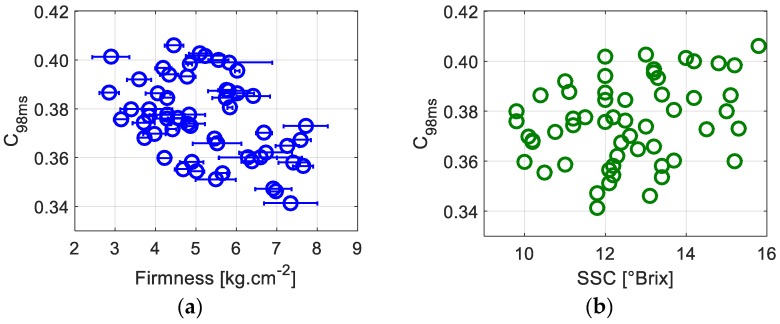
The contrast computed over rings with a 9.2 inner and 9.4 mm outer diameter at an exposure duration of 98 ms (*C*_98ms_) as a function of the firmness (**a**) and (**b**) the SSC level. Horizontal error bars correspond to standard deviations of three different performed firmness measurements.

In order to verify the existence of a relationship between the dynamics assessed using speckle fluctuations and apples’ physiological parameters, we first plot (Figure 3) *C*_98ms_ as a function of the firmness and SSC level. Figure 3a shows the contrast at an exposure duration of 98 ms as a function of apple firmness. A negative correlation between the two variables is observed, mainly showing a lesser *C*_98ms_ for higher firmness: in other words, the biospeckle activity increases with the firmness. Previous works monitoring fruit quality during postharvest storage have already exhibited such trends [20,21,27]; however, we have to point out that, in our case, we have no prior knowledge of the harvest date and the fruits’ origins. In fact, during storage, the fruit loses its firmness and juiciness. Postharvest ripening and evolution towards senescence and mealiness are accompanied by water evaporation and free air storage. This could cause a decrease in the mobility of scattering centers, meaning less biospeckle activity and higher contrast values.

**Figure 4:**
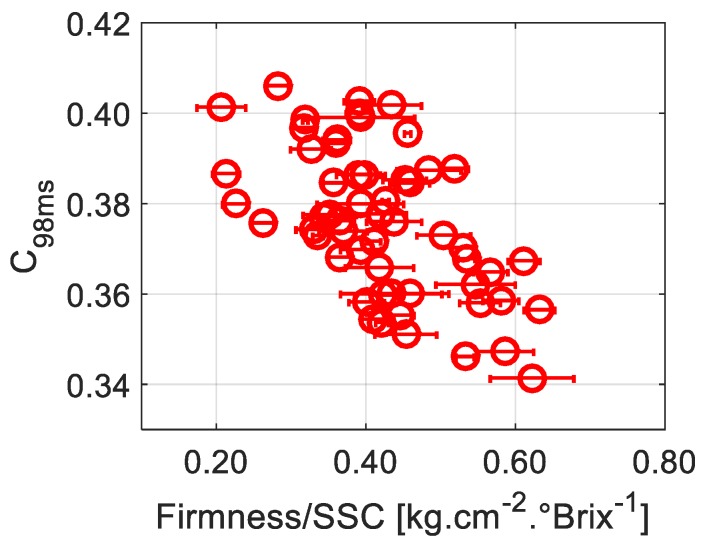
The contrast computed over rings with a 9.2 inner and 9.4 mm outer diameter at an exposure duration of 98 ms (*C*_98ms_) as a function of the ratio of the firmness to the SSC level. Horizontal error bars correspond to standard deviations of three different performed firmness measurements.

Moreover, during shelf life, it was shown that SSC slightly increases then stabilizes [28]. However, no study has found a significant correlation between this parameter and speckle activity to the best of our knowledge. Figure 3b, representing *C*_98ms_ as a function of the SSC, confirms the absence of significant correlation between these parameters. However, when considering *C*_98ms_ as a function of the ratio of the firmness to the SSC level, a better correlation can be seen in Figure 4 than that observed in Figure 3a, where we only take into account the firmness. This correlation was also highlighted with a false color scale in Figure 2. This suggests that the activity of biospeckle is not only related to firmness but also to the SSC level. Indeed, the SSC is directly connected to starch degradation. As starch granules are hydrolyzed into simpler carbohydrates and sugar molecules, the SSC increases. The contribution of SSC in the change of contrast levels, or biospeckle activity, could suggest that the soluble sugars contribute to the scattering process. This contribution has not yet been clearly elucidated [16]; given that a biological material is very complex with a multitude of phenomena occurring at the same time, in most cases it is hard to straightforwardly isolate the causes of the observed optical dynamics.

The assessment of Pearson’s correlation matrix is a common practice to reveal a relationship between different variables. Correlations between conventionally and optically measured parameters for the whole batch of apples are presented in Table 1 showing Pearson’s correlation coefficients. Note that the correlation coefficient *R* of two variables is defined as the ratio of their covariance to the product of their standard deviations. The significance of the correlation coefficient *R* can be assessed using the *p* value, which represents the probability that the results from the sample data occurred by chance. Coefficients corresponding to significant correlations are highlighted in bold.

According to Table 1, the correlation between *C*_98ms_ and firmness is −0.43. This value is significant, taking into account the unknown origin of the studied fruits, and the fact that all apples were taken into account when calculating the correlation, even those that had a high firmness standard deviation. The correlation is increased to −0.58 when considering *C*_98ms_ and the firmness/SSC, proving the SSC’s influence, which is opposite to that of the firmness. During shelf life, as mentioned earlier, the firmness decreases, causing an increase in *C*_98ms_, or, in other words, a decrease in activity. In parallel, the SSC tends to increase, causing an increase in *C*_98ms_. On the one hand, higher firmness is associated with the presence of a higher amount of water, possibly allowing higher mobility of scattering centers. On the other hand, greater SSC levels could cause a reduction in scatterers’ mobility due to an increase in internal fluids’ viscosity. This means that during shelf life both parameters will have made positive contributions to the biospeckle activity. This could explain the good correlation that was observed in previous works [20] between the number of days of storage and biospeckle activity, knowing that the firmness decreases while the SSC tends to increase.

The parameters retrieved from linear adjustments applied to contrast profiles over shorter and longer exposures (slopes *a*_short_ and *a*_long_ and intercepts *b*_short_ and *b*_long_) were also compared to conventional measurements. No correlation between |*a*_long_| and neither of the parameters was found. However, significant correlation values were obtained when comparing the firmness to each of the parameters: |*a*_short_|, *b*_short_, and *b*_long_ (correlation coefficient *R* = 0.44, −0.35, and −0.43, respectively). All of these correlations were improved when considering the firmness/SSC instead of only the firmness (see Table 1).

**Figure 5:**
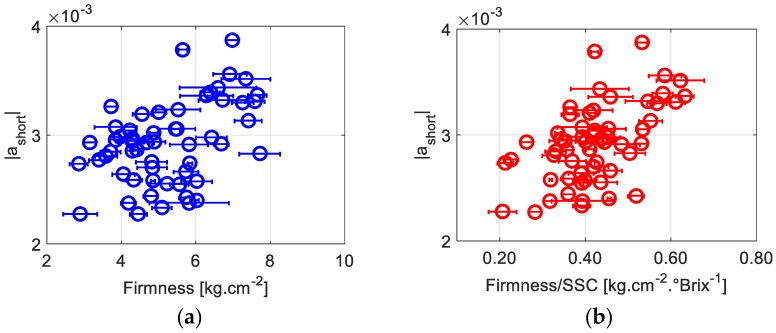
The speed of contrast degradation at short exposure durations |*a*_short_| as a function of the firmness (**a**) and (**b**) the ratio of the firmness to the soluble solids content (SSC). Horizontal error bars correspond to standard deviations of three different performed firmness measurements.

The parameter |*a*_short_|, describing the speed by which the contrast decreases at shorter exposures, is particularly more correlated to Firmness/SSC than to Firmness only (*R* = 0.55 vs. 0.44). Figure 5a,b display the evolution of |*a*_short_| as a function of firmness and firmness/SSC. Observably, |*a*_short_| is high for higher firmness and lower SSC values. These results show that the influence of the firmness and SSC level on biospeckle activity starts happening at very short time scales, meaning that the involved dynamics are quite fast, on the order of a few milliseconds.

**Figure 6:**
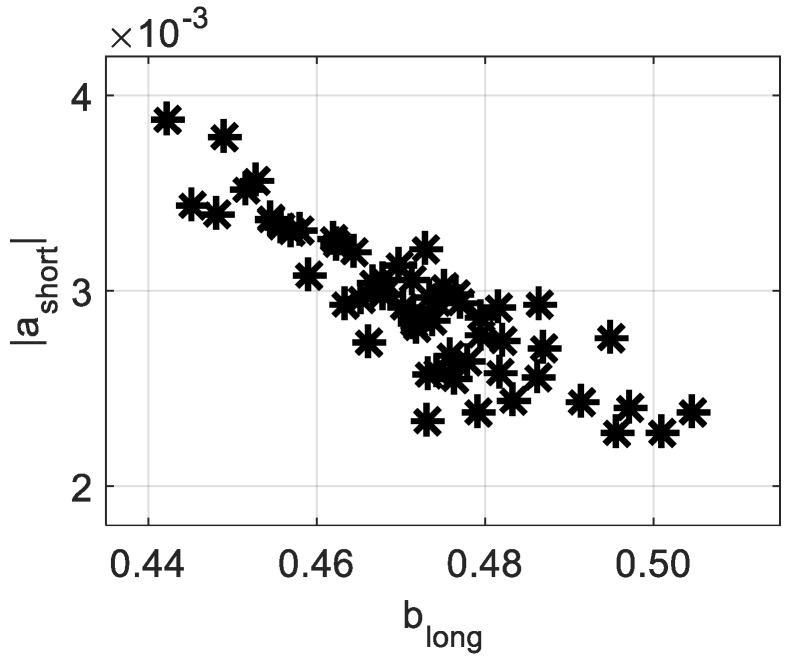
|*a*_short_| as a function of *b*_long_ for all studied apples.

Another highly significant correlation was found between *a*_short_ and *b*_long_ (*R* = −0.87). Examining this relationship displayed in Figure 6, one could broadly presume a relationship between the activity over shorter and longer exposures. However, at this point, any stated assumption about the basis of this relationship would be crude and would lack enough evidence. Further investigations should be performed in order to clarify the physical or biological grounds of such results.

## 4. Conclusions

The present study gives new insight into biospeckle activity in apple tissues. Light scattering by apple flesh was investigated via scattering spot images acquired at different exposure durations using a very simple optical setup. The existence of biospeckle activity at very short time scales, on the order of a few milliseconds, has been confirmed. This highlights the presence of fast dynamics detectable via speckle intensity fluctuations, thus light scattering by rapidly diffusing constituents. Despite the use of apples of an unknown origin, previous storage conditions, and earlier shelf life, a correlation between the assessed rapid activity and apples’ physiological parameters was found. Fast activity-related parameters (contrast values at an exposure time of 98 ms, and slopes of the contrast as a function of time at short exposures) correlated rather well with apple firmness. The correlation was enhanced when considering the ratio of the firmness to the SSC level. Those results suggest a relationship between the high speed inner activity and apple tissue firmness, as well as sugar levels. This study therefore gives insight about relevant time scales for apples’ optical diagnosis.

## Figures and Tables

**Table 1:** Correlation coefficients calculated for *C*_98ms_, |*a*_short_|, *b*_short_, |*a*_long_|, *b*_long_, firmness, soluble solids content (SSC), and firmness/SSC measured for the whole batch of apples. Values in bold indicate a significant correlation at *p* < 0.01.

	*C*_98ms_	|*a*_short_|	*b*_short_	|*a*_long_|	*b*_long_	Firmness	SSC	Firmness/SSC
*C*_98ms_	1	-	-	-	-	-	-	-
|*a*_short_|	**−0.88**	1	-	-	-	-	-	-
*b*_short_	**0.61**	**−0.78**	1	-	-	-	-	-
|*a*_long_|	**−0.55**	0.19	0.06	1	-	-	-	-
*b*_long_	**0.68**	**−0.87**	**0.76**	0.23	1	-	-	-
Firmness	**−0.43**	**0.44**	**−0.35**	0.09	**−0.43**	1	-	-
SSC	0.29	−0.22	0.17	−0.25	0.12	0.26	1	-
Firmness/SSC	**−0.58**	**0.55**	**−0.41**	0.23	**−0.48**	**0.88**	−0.22	1
